# Oral Exposure to Tributyltin Induced Behavioral Abnormality and Oxidative Stress in the Eyes and Brains of Juvenile Japanese Medaka (*Oryzias latipes*)

**DOI:** 10.3390/antiox10111647

**Published:** 2021-10-20

**Authors:** Yanhong Shi, Chen Chen, Ming Li, Lei Liu, Kejun Dong, Kun Chen, Xuchun Qiu

**Affiliations:** 1 Institute of Environmental Health and Ecological Security, School of the Environment and Safety Engineering, Jiangsu University, Zhenjiang 212013, China; shiyanhong2003@126.com (Y.S.); chenchen9688@yeah.net (C.C.); liming@ujs.edu.cn (M.L.); llleiliu@163.com (L.L.); dkjcl0516@163.com (K.D.); 2 Jiangsu Collaborative Innovation Center of Technology and Material of Water Treatment, Suzhou University of Science and Technology, Suzhou 215009, China

**Keywords:** tributyltin, Japanese medaka, behavior, oxidative stress, ATPase

## Abstract

The widely used compound tributyltin (TBT), which can be continuously detected in aquatic species and seafood, may induce diverse adverse effects on aquatic organisms. However, little is known regarding the mechanistic links between behavioral abnormality and oxidative stress in different fish tissues in response to oral TBT exposure. Herein, juvenile Japanese medaka (*Oryzias latipes*) were orally exposed to TBT at 1 and 10 ng/g-bw/d for four weeks. After exposure, the locomotor activity and social interaction of juvenile medaka were found to be significantly reduced in the 10 ng/g-bw/d TBT-exposed group. Furthermore, the antioxidant biomarkers in different tissues of juvenile medaka showed different levels of sensitivity to TBT exposure. The eye superoxide dismutase (SOD) activities markedly increased in both groups exposed to 1 and 10 ng/g-bw/d TBT, while the eye and brain malondialdehyde (MDA) levels increased in the higher dose group. Furthermore, the eye and brain ATPase activities markedly declined in the 1 ng/g-bw/d TBT-exposed group. A correlation analysis revealed that the decreased locomotor activity and social interaction in medaka were associated with the eye antioxidant enzyme (i.e., SOD and catalase (CAT)) activity and brain oxidative damage level. Thus, our findings suggested that there might be some mechanistic links between the behavioral abnormality induced by TBT exposure and oxidative stress in the eyes and brains of medaka. Thus, our findings indicate that the impacts of oral exposure to TBT should be considered to better assess its risk to the aquatic ecosystem and human health.

## 1. Introduction

Tributyltin (TBT) is an organotin that has been widely used as an antifouling agent since the early 1960s. Due to its high toxicity to aquatic organisms, many countries have controlled or banned the use of TBT antifouling paints since the 1980s. For example, TBT-containing antifouling paints were banned on boats < 25 m in France, the US, and Canada, and such paints were also banned from use in fish farming nets in the UK and Japan. Furthermore, Germany banned the sale and use of TBT-containing antifouling paints [1]. Finally, the use and production of TBT-containing antifouling paints have been strictly banned in most countries worldwide since 2003 [2,3]. At present, TBT is only used in some developing countries. However, due to its high efficacy in adsorbing sediments, the half-life of TBT ranges from hundreds of days in estuarine sediments to tens of years in anaerobic sediments [4]. Moreover, high levels of TBT have continued to be detected in different tissues of aquatic species and seafood [5,6,7]. Thus, oral intake is still an important route of TBT exposure for both aquatic species and humans.

As an endocrine-disrupting chemical, numerous studies have been indicated that TBT could induce reproductive toxicity in fish. For example, exposure to environmentally relevant concentrations of TBT could decrease the hatching viability of the eggs of Japanese whiting (*Sillago japonica*) [8] and delay the oogenesis of cuvier (*Sebasticus marmoratus*) [9]. Exposure to 5.8 µg/L of tributyltin oxide (TBTO) could reduce the counts of spermatids due to the inhibition of the proliferation of spermatogenic cells in mummichog (*Fundulus heteroclitus*) [10]. Masculinization has also been observed in several fish species [10,11,12] and other aquatic organisms [13] during their developmental stages after exposure to TBT. At the same time, exposure to TBT has also been found to induce abnormal development in the embryos of medaka (*Oryzias latipes*) [14] and decrease the developmental success of Manila clam (*Ruditapes philippinarum*) embryos [15]. In addition, recent studies have shown that exposure to TBT could depress the predatory activity of *Sebastiscus marmoratus* [16], enhance aggressive behavior and fear response in adult zebrafish (*Danio rerio*) [17], and reduce locomotor activity in zebrafish larvae [18]. Changes in neurotransmitter levels are generally associated with altered fish behavior in responses to TBT exposure, but some unclear mechanisms are involved in its behavioral toxicity [18,19].

It is currently accepted that oxidative stress may trigger the occurrence and development of various diseases, including behavioral impairments [20,21,22]. In mammals, it has been proven that the oral administration of TBT could induce neuronal damage, with oxidative stress and neuroinflammation being common features that may lead to subsequent behavior impairments [22,23]. In teleosts, previous studies have also reported that TBT could induce oxidative stress in different tissues. For example, Zhang et al. [24] found that exposure to TBT (10 and 100 ng/L for 56 days) decreased the superoxide dismutase (SOD) and catalase (CAT) activities and increased the malondialdehyde (MDA) content in the liver of zebrafish. Furthermore, Li et al. [25] reported that the MDA content significantly increased in the intestine of juvenile common carp exposed to TBT at a level of 7.5 µg/L for 60 days. Nevertheless, no report so far has focused on the mechanistic links between behavioral abnormality and oxidative stress in fish tissues in response to oral TBT exposure. In addition, TBT exposure could disturb the energy metabolism in fish species, leading to subsequent behavior impairments [26,27,28].

Therefore, we exposed Japanese medaka (*Oryzias latipes*) juveniles to TBT via their diet at doses of 0, 1, and 10 ng/g body weight per day (ng/g-bw/d). After four weeks of exposure, changes in the behavioral traits of medaka were investigated. Subsequently, variations in the levels of antioxidant indicators (SOD, CAT, and glutathione (GSH)), an oxidative damage indicator (MDA), and total ATPase were examined in homogenates of the eyes and brain. Our main objective was to explore the possible mechanistic links between behavioral abnormalities and oxidative stress in the eyes and brain of medaka in response to oral TBT exposure.

## 2. Materials and Methods

### 2.1. Organisms

Japanese medaka juveniles (four weeks post-hatching, initial body weight: 37.7 ± 6.3 mg) were cultured in several 20 L cylindrical glass tanks containing 15 L of artificial seawater (temperature = 27 ± 1 °C; salinity = 1‰; half of the volume was changed every two days). The fish were kept under a 14:10 h light:dark cycle and fed with dry food (*Artemia nauplii*, Weifang Yee Pet Products Co., Ltd. (Weifang, Shandong, China)) at a dose of 1% of the individual’s body weight per day.

### 2.2. Chemicals and Preparation of Test Diets

Tributyltin chloride was purchased from Dr Ehrenstorfer GmbH (Augsburg, Germany). Other reagents (analytical grade) were purchased from Sinopharm Chemical Reagent Co., Ltd. (Shanghai, China). Enzyme-linked immunosorbent assay (ELISA) kits for SOD, CAT, GSH, MDA, and ATPase were purchased from Bomei Biotechnology Co., Ltd. (Hefei, Anhui, China). Test diets containing TBT were prepared according to the method described by Qiu et al. [29]. Briefly, the TBT solution was prepared by dissolving it into ethanol at concentrations of 0, 1, or 10 μg/mL. Then, 0.1 mL of the 0, 1, and 10 μg/mL TBT solutions were added and mixed with 1 g of dry food (*Artemia nauplii*). The final TBT concentrations in the dry food were 0 (control), 0.1, or 1 μg/g-diet for the different exposure groups. All the prepared diets were dried at 35 °C overnight and stored at 4 °C until use. In previous studies, TBT was detected at levels from 0.001 to 2.10 μg/g (μg Sn per g dry weight) in the sediment of the port of Osaka [30]; 0.0024 to 8.548 μg/g in the fishing harbors of Taiwan, China [31]; and in bivalves at levels from 0.008 to 0.135 μg/g in Northern Kyushu, Japan [15]. Therefore, the concentrations of TBT used in the present study are environmentally relevant levels that have been detected in sediments and aquacultures.

### 2.3. Exposure Test

Three experimental groups were cultured in three tanks containing 35 healthy juveniles each. The fish were fed with prepared diets containing TBT at levels of 0, 0.1, and 1 μg/g-diet with a dose of 1% of the individual’s body weight per day. In this way, the exposure dosages of TBT were 0, 1, or 10 ng/g-bw/d. The three treatment groups were accordingly named control, TBT-1, and TBT-10, respectively. The exposure experiment was conducted for four weeks under the same conditions as mentioned in Section 2.1, and the survival and abnormal development of juvenile medaka were monitored daily. In addition, after the 4-week exposure, behavioral traits were tracked. Subsequently, all the fish were sampled and immediately frozen on ice. Then, the eyes and brains of the fish were freshly excised, weighed, and stored at −80 °C until the biochemical analysis.

### 2.4. Behavioral Traits Measurement and Analysis

The behavior of medaka was recorded during the same time interval (10:00 to 12:00 China standard time) to minimize the possible effects of diel periodicity [32]. Eight fish were randomly selected for each treatment and placed in four circular glass aquariums (9 cm diameter and 6 cm height; two fish in each, *n* = 4) containing 60 mL of artificial seawater. After a 10 min acclimation period, fish locomotion was tracked for 10 min using a Danio Vision system (Noldus, Wageningen, The Netherlands) and analyzed using the EthoVision XT software (Vison 11.5; Noldus). Parameters representing behavioral traits were measured and calculated according to the descriptions provided by Qiu et al. [33].

### 2.5. Biochemical Assays

The activities of SOD, CAT, and ATPase and contents of GSH and MDA in the eyes and brains of juvenile medaka were determined in order to evaluate the oxidative stress of medaka given the different treatments in different tissues. Tissues from two fish were pooled as one replicate, and there were six replicates for each treatment group. The tissue homogenate and supernatant for the biological assays were prepared following the method described by Wu et al. [32]. Briefly, each sample was weighed and homogenized with 9 vol (*w*/*v*) of phosphate buffered saline (10 mM, pH 7.2–7.4). Then, the homogenate was centrifuged at 6000× *g* for 5 min and the supernatant was collected for further use. The obtained supernatant was used to measure the SOD, CAT, and ATPase activities; GSH and MDA contents; and protein concentrations in each tissue using the corresponding kit (Bomei Biotechnology Co., Ltd.) according to the manufacturer’s instructions. The activities of SOD, CAT, and ATPase and levels of GSH and MDA were expressed as IU or U per mg protein and pg per mg protein, respectively.

### 2.6. Statistical Analysis

As some data could not satisfy the assumptions of homogeneity of variance even after logarithmic transformation, a generalized linear model (GzLM) was employed to analyze the statistical significance (*p* < 0.05) in behavioral traits and biochemical parameters across the three treatments. The correlations of behavioral traits with biochemical parameters (the corresponding values obtained from the same sample) were tested for significance (*p* < 0.05) using Spearman’s correlation analysis. All statistical analyses were performed using the SPSS Advanced Models 11.0J software (SPSS Japan, Tokyo, Japan). Microsoft Excel 2016 and OriginPro 9.2 (Origin Lab Corporation, Northampton, MA, USA) were used for the generation of plots.

## 3. Results

No mortality was recorded during the experiment period. After the 4-week exposure, the average body weights of medaka were found to be 76.3 ± 15.9, 79.1 ± 12.3, and 70.1 ± 17.7 mg in the control, TBT-1, and TBT-10 groups.

### 3.1. Responses in the Behavioral Parameters Related to Locomotor Activity

**Figure 1:**
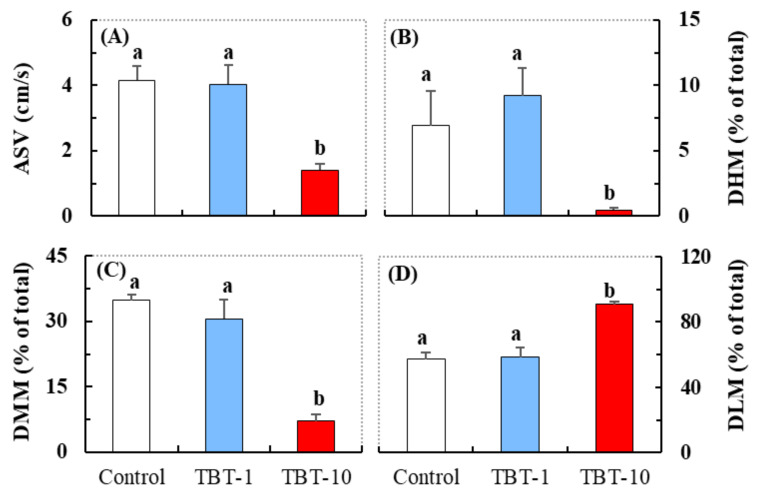
The average swimming velocity (ASV, **A**) and cumulative duration of high mobility (DHM, **B**), moderate mobility (DMM, **C**), and low mobility (DLM, **D**) activity of medaka exposed to TBT at the level of 0 (control), 1 (TBT-1), and 10 (TBT-10) ng/g-bw/d. Data are shown as mean ± SD (*n* = 4); values that do not share a common letter are significantly different at *p* < 0.05.

Compared with the control, a significantly reduced average swimming velocity (ASV), cumulative duration of high mobility (DHM), and cumulative duration of moderate mobility (DMM) were detected in medaka in the TBT-10 group, but not in the TBT-1 group (Figure 1A–C). A significantly elevated cumulative duration of low mobility (DLM) was also detected in medaka in the TBT-10 group (Figure 1D). Depending on the changing relative to the two defined thresholds of mobility [33,34], the locomotor activity of medaka was divided into three states (i.e., high mobility, >60%; moderate mobility, 20–60%; low mobility, <20%). Thus, the above behavioral responses suggested that oral exposure to TBT at a level of 10 ng/g-bw/d could induce hypoactivity in medaka.

### 3.2. Responses in the Behavioral Parameters Related to Social Interaction

**Figure 2:**
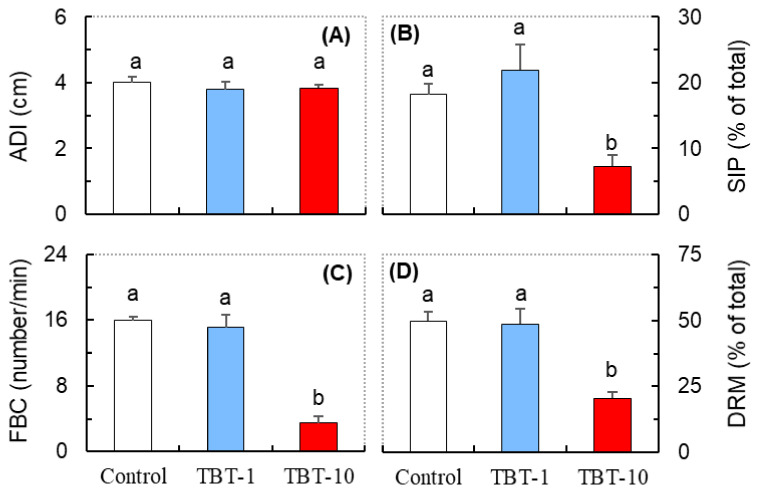
The average distance (ADT, **A**), state in proximity (SIP, **B**), frequency of body contact (FBC, **C**), and cumulative duration of relative movement (DRM, **D**) in medaka exposed to TBT at levels of 0 (control), 1 (TBT-1), and 10 (TBT-10) ng/g-bw/d. Data are shown as mean ± SD (*n* = 4); values that do not share a common letter are significantly different at *p* < 0.05.

Compared with the control, there were no significant changes in the average distance travelled (ADI) between the medaka exposed to either dose of TBT (Figure 2A). However, significant reductions in the state of proximity (SIP, where the distance between the selected body points of two subjects is lower than 1 cm), frequency of body contact (FBC), and cumulative duration of relative movement (DRM, where the state of two subjects is moving (towards or away) relative to each other) were detected in medaka in the TBT-10 group, but not those in the TBT-1 group (Figure 2B–D). Thus, oral exposure to TBT at a level of 10 ng/g-bw/d could significantly decrease the social interaction in medaka.

### 3.3. Responses in the Antioxidant Biomarkers and ATPase

**Figure 3:**
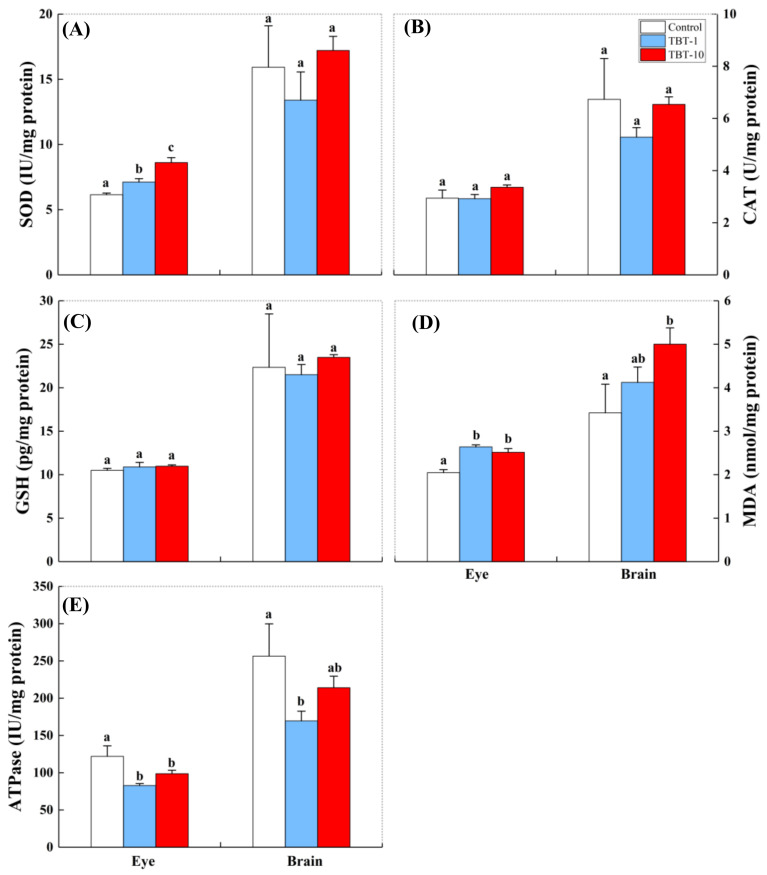
Variations in the levels of SOD (**A**), CAT (**B**), GSH (**C**), MDA (**D**), and ATPase (**E**) in different tissues (eyes and brain) exposed to TBT at 0 (control), 1 (TBT-1), and 10 (TBT-10) ng/g-bw/d. Data are shown as mean ± SD (*n* = 6); values that do not share a common letter are significantly different at *p* < 0.05.

As shown in Figure 3A–D, oral exposure to TBT induced tissue-specific oxidative stress in medaka. In the eyes, significantly increased SOD activity (Figure 3A) was observed in medaka given both doses of TBT, but the CAT activity (Figure 3B) and GSH content (Figure 3C) did not significantly alter in fish given either dose. In addition, the eye MDA content was only significantly increased in the TBT-10 group compared with the control group (Figure 3D). In the brain, the SOD activity and MDA content were only significantly increased in the TBT-10 group (Figure 3A,D), while the CAT activity and GSH content did not significantly change in fish given either TBT dose (Figure 3B,C).

As shown in Figure 3E, the ATPase activities in the eyes and brain exhibited a declining trend, but only significantly decreased in the TBT-1 group. However, a slight increase in the ATPase activity in the group given a higher dose of TBT exposure was observed in both the eyes and brain of medaka.

### 3.4. Correlations Analysis

As shown in Table 1, the correlations of biochemical parameters in the eye and brain samples with the behavioral traits of medaka exhibited different characteristics. For the eye samples, the SOD activity was negatively correlated with ASV (*p* < 0.05), DHM (*p* < 0.05), DMM (*p* < 0.01), SIP (*p* < 0.05), FBC (*p* < 0.01), and DRM (*p* < 0.01), while the CAT activity was also negatively correlated with DMM (*p* < 0.05), SIP (*p* < 0.01), and FBC (*p* < 0.05). Thus, the decreased locomotor activity and social interaction seen in medaka may be associated with increased oxidative stress in the eyes. For the brain samples, only the MDA content exhibited a significant negative correlation with DHM (*p* < 0.05), DMM (*p* < 0.05), SIP (*p* < 0.01), and FBC (*p* < 0.01). Thus, the decreased locomotor activity and social interaction in medaka may be associated with brain oxidative damage levels. Moreover, the behavioral trait of DLM exhibited significant positive correlations with the eye SOD and CAT activities and the brain MDA content.

## 4. Discussion

Our results demonstrated that oral exposure to TBT at a level of 10 ng/g-bw/d induced hypoactivity in juvenile medaka, as indicated by their significantly decreased ASV, DHM, and DMM values (Figure 1). Consistent with our results, previous studies have demonstrated that contact with TBT or other organotin, mostly occurring through waterborne exposure, could alter the locomotor activity of various fish species [2,17,35]. For example, Schmidt et al. [36] reported that the swimming speed of carp (*Cyprinus carpio*) markedly declined after they were exposed to 7 µg/L of TBT for 4–6 days, while Liang et al. [18] also detected hypoactivity in zebrafish larvae exposed to 1 nM of TBT for four days. Furthermore, hypoactivity in fish induced by chemical exposure has been associated with lower levels of ingestion, weakened ability to avoid predators, and reduced chances of reproduction [37,38]. Thus, we inferred that the reduced locomotor activity induced by oral TBT exposure might affect the fitness of fish in their natural environment.

We also found that exposure to TBT at the level of 10 ng/g-bw/d disturbed the social interactions of medaka, as indicated by the significantly reduced SIP, FBC, and DRM (Figure 2). Consistent with our results, Zhang et al. [39] reported that rare minnow (*Gobiocypris rarus*) exposed to TBT exhibited a shorter latency before leaving their shoal mates and spent more time away from the shoal, while Xiao et al. [40] found that TBT significantly suppressed the reproductive behaviors of zebrafish. Since medaka is a shoaling fish and the females prefer to mate with socially familiar males, we inferred that the inhibited social interaction induced by TBT might further affect their mating behavior and chances of reproductive success. Indeed, previous studies have demonstrated that TBT decreased reproductive success in zebrafish and Japanese medaka [40,41].

Changes in neurotransmission and hormone secretion are generally considered critical factors of the behavioral impairments induced by TBT exposure [17,39,40]. Nevertheless, recent studies have also suggested that some other mechanisms, such as neuronal damage caused via inducing brain oxidative stress, may also be involved in the behavioral toxicity of TBT [18,22,23]. Meanwhile, previous studies have indicated that exposure to TBT could lead to lesions in the eyes and brains of fish larvae and juveniles, such as corneal keratitis and increased vacuolation in guppies and medaka [42], as well as alterations in the cellular and fibrillar structures of the brain in larvae [1], suggesting that the eyes and brains of fish are important target organs of TBT. Therefore, we investigated whether the behavioral changes observed are related to oxidative stress in the eyes and brains of medaka under TBT stress, and, if so, which oxidative stress biomarkers are involved.

We observed that the activities of eye antioxidant enzymes were susceptible to TBT exposure. Furthermore, the dose-dependent increases in SOD activity suggested that oral exposure to TBT induced ROS production in fish eyes. At the same time, the elevated MDA content further increased oxidative damage in the eyes of medaka. Similarly, Kim et al. [43] found that trimethyltin (an organotin) exposure could induce ROS-mediated apoptosis in zebrafish retinal cells. Moreover, TBT exposure was shown to cause degenerative alterations in the corneal epithelium, lens, retina and pigment layer of the retina, and choroid of minnow *Phoxinus phoxinus* larvae by histological analysis, finally resulting in the opaque color of the eyes [44]. Bruno and Ellis [45] also found an increase in the eye opacities of the Atlantic salmon (*Salmo salar*) after they were exposed to TBT-treated nets for seven weeks. Guo et al. [46] observed abnormal eyes in *Xenopus tropicalis* embryos, including the loss of eye pigmentation or the absence of external eyes, after exposure to environmentally relevant TBT concentrations for 24 to 48 h. The eye is a key sensory system in fish due to its role in collecting and focusing images and transforming them into neural signals. However, only a few studies have investigated the association between eye damage and other impacts in fish in response to environmental pollution [47,48]. For example, chlorpyrifos could alter the behavioral responses of medaka to stimuli via inhibiting the acetylcholinesterase activity in their eyes [48]. Our results demonstrated for the first time that the eye SOD and CAT activities induced by TBT were negatively associated with the locomotor activity and social interaction of medaka, highlighting fish eyes as a critical target organ for environmental health assessment.

In the brain, the levels of CAT and GSH were not significantly altered by TBT (Figure 3B–C). However, the significantly increased SOD and MDA levels suggested that elevated oxidative damage occurred in the brain of medaka orally exposed to TBT at a level of 10 ng/g-bw/d. Similarly, a previous study by Li et al. [49] observed that exposure to sublethal concentrations of (5, 10, and 20 µg/L) of TBT for seven days resulted in significantly higher levels of oxidative indices (MDA) in the brains of juvenile common carp (*Cyprinus carpio*). Zhang et al. [50] showed that exposure to 1, 10, and 100 ng/L of TBT induced apoptosis in brain cells and increased reactive oxygen species in the brain of female *Sebastiscus marmoratus*. Moreover, a correlation analysis showed that several behavioral parameters were negatively correlated with the brain MDA levels (Table 1), suggesting that the elevated oxidative damage sustained in the brain may contribute to hypoactivity and lower levels of social interaction in response to TBT, which are detrimental to the survival of fish in the aquatic environment. In addition, the concentrations of TBT accumulated in medaka need to be measured in future studies to validate whether the changes in antioxidant enzyme activities and MDA levels were correlated with accumulated TBT in the eyes and brains of medaka.

Our results also revealed that TBT exposure significantly reduced the ATPase activity in the TBT-1 group in both the eyes and brains of medaka (Figure 3E). Similarly, Zhang et al. [51] reported that TBT exposure (0.1, 1, and 10 ng/L for 144 h) reduced the activities of Ca^+^-ATPase in the brains of rockfish (*Sebastiscus marmoratus*), while Li et al. [49] detected a decrease in Na^+^-K^+^-ATPase activities in the brains of juvenile common carp (*C. carpio*) exposed to TBT at a level of 20 µg/L for seven days. Interestingly, a slight increase in the ATPase activity (in both the eyes and brain, Figure 3E) was observed in the TBT-10 group compared with the TBT-1 group. Similar to this finding, a previous study reported that the ATPase activities in the gills and mantle of *Mytilus galloprovincialis* decreased, then increased, and finally decreased again with gradually increasing TBT doses [52]. Thus, the effect of TBT on ATPase activity in tissues may not always be concentration-dependent. Although we did not detect significant correlations between ATPase activity (in the eyes and brain) and behavioral traits, it cannot be concluded that deficiencies in energy metabolism do not affect observed behavioral abnormalities. ATPase is an enzymatic family that is involved in energy metabolism and responsible for many physiological activities [53]. As one of the most energetically demanding systems in the brain, deficiencies in energy metabolism in the visual system can lead to retinopathy, visual deficits, neuronal degeneration, and eventual blindness [54]. Thus, the lower levels of ATPase activity induced by TBT may play an indirect role in affecting the behavioral traits of medaka via disturbing the physiological function of the eyes and brain.

## 5. Conclusions

In conclusion, TBT induced oxidative damage in the eyes and brain of fish in addition to its previously reported reproductive toxicity. This oxidative damage may ultimately affect fish behaviors, such as locomotor activity and social interactions. The hypoactivity induced by TBT in fish has been associated with lower levels of ingestion and a weaker ability to avoid predators. These changes affect fish populations and the balance of the entire aquatic ecosystem [37,38]. Meanwhile, exposure to TBT was shown to disturb the social interactions of fish, which may suppress their reproductive behaviors and further affect their reproductive success [40,41]. As TBT can still be continuously detected in aquatic species and seafood [5,6,7], our findings highlight that the impacts of oral exposure to TBT should be considered to better assess its risk to the aquatic ecosystem and human health. It should also be noted that only eight fish were used for behavioral tests, meaning that these may not be a good representation of the behavioral responses of juvenile medaka to TBT oral exposure. Therefore, to validate the results from this study, more fish should be included in behavioral analyses in future studies.

## Figures and Tables

**Table 1:** Spearman’s correlation between the level of oxidative stress biomarkers, ATPase, and behavioral parameters of juvenile medaka exposed to TBT.

Spearman’s Coefficients ^1^	Eye					Brain				
	SOD	CAT	GSH	MDA	ATPase	SOD	CAT	GSH	MDA	ATPase
Behavioral parameters related to locomotor activity										
ASV	−0.643 *	−0.538	−0.491	−0.042	−0.175	−0.469	−0.371	−0.392	−0.573	−0.273
DHM	−0.594 *	−0.559	−0.456	0.105	−0.287	−0.573	−0.483	−0.441	−0.587 *	−0.42
DMM	−0.818 **	−0.636 *	−0.47	−0.301	0.028	−0.329	−0.056	−0.371	−0.622 *	0.007
DLM	0.636 *	0.594 *	0.448	−0.028	0.252	0.503	0.343	0.441	0.580 *	0.301
Behavioral parameters related to social interaction										
ADI	−0.077	0.231	−0.488	−0.189	0.203	0.056	0.028	0.161	0.042	0.112
SIP	−0.692 *	−0.671 *	0.021	−0.084	−0.098	−0.364	−0.154	−0.399	−0.727 **	−0.182
FBC	−0.741 **	−0.608 *	−0.137	−0.210	−0.021	−0.406	−0.287	−0.315	−0.769 **	−0.196
DRM	−0.622 *	−0.531	−0.456	0.021	−0.217	−0.448	−0.343	−0.357	−0.524	−0.287

^1^ The detailed abbreviations for behavioral parameters and oxidative stress biomarkers are shown in Section 3.1 and Section 3.2; asterisks indicate statistical significance (* *p* < 0.05; ** *p* < 0.01). Only the biochemical parameters obtained from the brain and eye samples collected from fish participating in the behavioral test were used for the correlation analysis (i.e., *n* = 4 for each treatment).

## Data Availability

Data is contained within the article.
